# HER2-Targeted Antibody–Drug Conjugates Display Potent Antitumor Activities in Preclinical Extramammary Paget’s Disease Models: In Vivo and Immunohistochemical Analyses

**DOI:** 10.3390/cancers14143519

**Published:** 2022-07-20

**Authors:** Keiko Tokuchi, Takuya Maeda, Shinya Kitamura, Teruki Yanagi, Hideyuki Ujiie

**Affiliations:** Department of Dermatology, Faculty of Medicine and Graduate School of Medicine, Hokkaido University, Sapporo 060-8638, Japan; shibakei1017@yahoo.co.jp (K.T.); mtakun.bsb.hkt@gmail.com (T.M.); keiba1081@yahoo.co.jp (S.K.); h-ujiie@med.hokudai.ac.jp (H.U.)

**Keywords:** antibody–drug conjugate, HER2, *ERBB2*, extramammary Paget’s disease, patient-derived xenograft

## Abstract

**Simple Summary:**

The prognosis for advanced Extramammary Paget’s disease (EMPD) is almost always poor. HER2-targeted antibody–drug conjugates (ADCs) such as trastuzumab emtansine and trastuzumab deruxtecan have proven to be effective against HER2-positive breast cancers; however, no studies have addressed HER2-targeted ADCs as treatments for EMPD. We examine the efficacy of ADCs against an EMPD patient-derived xenograft (PDX) model harboring pathogenic *ERBB2* mutations. Treatment with trastuzumab emtansine or trastuzumab deruxtecan was found to significantly regress EMPD-PDX tumors in only seven days, with no recurrence observed for 10 weeks. Our results suggest that HER2-targeted ADCs could be novel and promising treatment options for patients with EMPD, especially in cases with the *ERBB2*-mutation or *ERBB2*-overexpression.

**Abstract:**

Extramammary Paget’s disease (EMPD) is an adenocarcinoma that develops mainly in the genital region of older adults. The prognosis for advanced EMPD is almost always poor; thus, novel therapeutic strategies need to be developed. HER2-targeted antibody–drug conjugates (ADCs) such as trastuzumab emtansine and trastuzumab deruxtecan have proven effective against HER2-positive breast cancers; however, no studies have addressed HER2-targeted ADCs as treatments for EMPD. We examine the efficacy of ADCs against an EMPD patient-derived xenograft (PDX) model harboring pathogenic *ERBB2* mutations and investigate the expression levels of HER2 using EMPD clinical samples. Trastuzumab emtansine or trastuzumab deruxtecan was administered intravenously to tumor-bearing NOD/Scid mice. Treatment with trastuzumab emtansine or trastuzumab deruxtecan was found to significantly regress EMPD-PDX tumors in only seven days, with no recurrence observed for 10 weeks. EMPD tumors extracted 48 h after drug administration revealed the TUNEL-positive ratio to be significantly higher for the HER2-targeted ADC-treated tumors than for the control tumors. EMPD patients’ clinical samples revealed a significant correlation between HER2 positivity and invasion, suggesting that HER2 status is associated with tumor progression. Our results suggest that HER2-targeted ADCs could be novel and promising treatment options for patients with EMPD, especially in *ERBB2*-mutant or *ERBB2*-overexpressed cases.

## 1. Introduction

Extramammary Paget’s disease (EMPD) is a rare cutaneous adenocarcinoma that is commonly seen in the genital epithelia of older adults [1]. Most EMPD cases are diagnosed as carcinoma in situ; thus, the prognosis is relatively favorable with surgical resection. However, once metastasis occurs, the prognosis becomes poorer. A multi-center retrospective study revealed the 5-year survival rate for EMPD patients with distant metastasis to be only 7% [2]. Although several chemical regimens such as docetaxel monotherapy and low-dose 5-fluorouracil/cisplatin therapy have been proposed for the treatment of advanced EMPD, their efficacy has proven limited [3]. Therefore, novel therapies for advanced EMPD need to be developed.

Human epidermal growth factor receptor 2 (HER2) is a 185-kDa receptor tyrosine kinase encoded by the *ERBB2* gene on chromosome 17q12. Upon ligand binding, HER2 activates downstream signaling cascades, such as the PI3K and MAPK pathways. *ERBB2* amplification causes HER2 overexpression, resulting in ligand-independent homo- or heterodimerization and abnormal downstream signaling activation [4]. Approximately 15–25% of breast cancers have been reported to be HER2-positive and correlate with more aggressive features [5,6]. Reportedly, the positive expression of HER2 has been observed in 15–60% of EMPD cases [7,8]. Given EMPD’s biological resemblance to breast cancers [3,7,8], HER2 is recognized as a crucial therapeutic target in HER2-positive EMPD patients.

Antibody–drug conjugates (ADCs) are monoclonal antibodies connected to cytotoxic agents. They make use of antibodies that are specific to tumor cell-surface transmembrane proteins; thus, they have tumor specificity and potency while minimizing toxicity in normal tissue. The mechanism of ADCs involves the recognition and connection of the monoclonal antibody backbone to the extracellular domain of cancer-specific membrane proteins, the internalization of the ADC-antigen complex via receptor-mediated endocytosis, and the intracellular release of the cytotoxic payload that induces cell death [9]. Two ADCs have been approved as treatments for HER2-positive breast cancer: trastuzumab emtansine (T-DM1) and trastuzumab deruxtecan (T-DXd) [10]. T-DM1 has the HER2-targeted antitumor properties of trastuzumab conjugated with the cytotoxic activity of the microtubule-inhibitory agent DM1 [10]. T-DXd is an ADC of trastuzumab and a cytotoxic agent exatecan derivative that is a topoisomerase I inhibitor [11]. ADCs provide a wider selection of therapeutic options and greater efficacy of chemotherapy in breast cancer [12]. Concerning anti-HER2 treatments for EMPD, Vornicova et al. reported the HER2-targeted drug lapatinib to be effective against HER2-positive EMPD [13]. Further, several case reports have shown the HER2-targeted antibody trastuzumab alone or with cytotoxic chemotherapies to be effective against metastatic or advanced EMPD in *ERBB2*-mutant or HER2/*ERBB2*-overexpressed cases (Supplemental Table S1) [14,15,16,17,18,19,20,21,22,23,24,25,26,27,28,29,30]. Notably, Nordmann et al. reported an EMPD tumor harboring the *ERBB2 S310F* mutation that was sensitive to anti-HER2 treatment, with the patient achieving a near-complete response by a combination treatment of trastuzumab and carboplatin [24]. Concerning anti-HER2 ADC, there has been only one case report in which the patient was treated with T-DM1 and complete remission was achieved [21]. For the preclinical models, we established an EMPD patient-derived xenograft (PDX) model harboring a pathogenic *ERBB2* S310F mutation against which anti-HER2 therapy and cytotoxic agents are effective [31]. Furthermore, in several other cancers, including lung cancer and colon cancers, HER2-targeted ADCs have proven effective in treating PDX models harboring pathogenic *ERBB2* mutations [32,33,34]. Based on the clinical and experimental results, we hypothesized that HER2-targeted ADCs could be effective treatments for EMPD. We investigated the efficacy of HER2-targeted ADCs in treating an EMPD-PDX model and assessed the protein expression of HER2 using EMPD clinical samples.

## 2. Materials and Methods

### 2.1. Reagents and Antibodies

Antibodies against HER2 were purchased from Dako (Code A0485, Hovedstaden, Denmark), and those against Ki-67 were purchased from Abcam (#ab8191, Boston, MA, USA). The T-DM1 (Kadcyla^®^) was purchased from Chugai Pharmaceutical/Roche (Tokyo, Japan). The T-DXd (Enhertu^®^) was purchased from Daiichi-Sankyo/AstraZeneca (Tokyo, Japan).

### 2.2. EMPD Patient-Derived Xenografts

We previously established PDXs from an EMPD tumor sample using Matrigel (BD Biosciences; Franklin Lakes, NJ, USA; EMPD-PDX-H1) [31]. Briefly, we transplanted tumor tissue from metastatic lymph nodes into immunodeficient NOD/Scid mice. The EMPD-PDX-H1 model showed similar morphology and protein expression to those of the patient’s primary tumor and metastatic lymph nodes [31]. All animal experiments were approved by the Institutional Animal Care and Use Committee of Hokkaido University (approval number 22-0034). NOD/Scid mice were purchased from Clea Japan. All animals used for the present study were maintained under pathogen-free conditions. The tumor-transplanted mice were observed twice a week, and the tumor volumes were measured twice a week by a caliper. Tumor volume was calculated using the following formula: (long axis × short axis^2^)/2 [35]. Once the tumor volume reached 500–1000 mm^3^, the EMPD-PDX-H1 tumor was passage into the next generation of NOD/Scid mice by subcutaneous transplantation.

### 2.3. Treatment Experiments Using HER2 Inhibitors and HER2-Targeted ADCs

**Figure 1:**
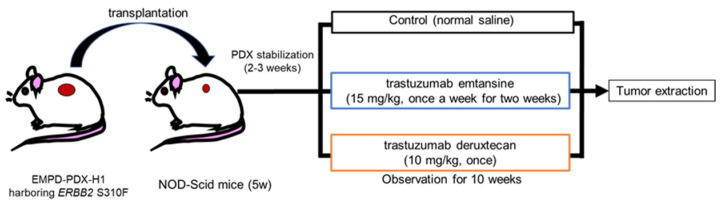
Schematic of treatment experiments with HER2-targeted ADCs using extramammary Paget’s disease patient-derived xenograft (EMPD-PDX) model mice. Tumor-bearing NOD/Scid mice were randomized into three groups. In the HER2-targeted treatments, trastuzumab emtansine (15 mg/kg) was administered intravenously once a week for two weeks, or trastuzumab deruxtecan (10 mg/kg) was administered intravenously once (n = 4, each group).

Tumor growth curves for all of the EMPD-PDX-H1 tumors were generated using kinetic measurements of tumor volume. The NOD/Scid mice bearing the PDX tumors (volume range: 50–100 mm^3^) were randomized into three groups, and treatment experiments were performed. The control mice were intravenously administered 100 μL PBS once a week (n = 4). For the HER2-targeted ADC treatments, T-DM1 (15 mg/kg) was administered intravenously once a week for two weeks (n = 4), or T-DXd (10 mg/kg) was administered intravenously once (n = 4), based on a previous study (Figure 1) [36]. Furthermore, to assess cell death in the tumors, we prepared 3 more NOD/Scid mice bearing the PDX tumors. They were sacrificed 48 h after the intravenous administration of PBS, T-DM1 (15 mg/kg), or T-DXd (10 mg/kg). We assessed the extracted tumors by Ki-67 staining and TUNEL assay as described in 2.4 and 2.5.

### 2.4. Histopathological Analyses

Histopathologically, in situ EMPD is defined as a malignant neoplasm confined to the squamous epithelium and adnexal tissues without invasion of the underlying tissues [37]. The degree of histological invasion is categorized as in situ/microinvasive (showing invasion until the papillary dermis) or invasive (showing invasion of the deep dermis) [38]. Immunohistochemical analyses were performed on 4 μm thick formalin-fixed, paraffin-embedded sections. Immunostaining was evaluated by the same observer. The expression levels of HER2 protein were evaluated in accordance with the US Food and Drug Administration-approved scoring guideline for breast carcinomas, i.e., the HercepTest scoring guideline: 0 for no staining or only membrane staining in <10% of the cells; 1+ for faint or barely perceptible staining of the incomplete cell membrane in >10% of the cells; 2+ for weak to moderate staining of the complete cell membrane in >10% of the cells; and 3+ for intense staining of the complete circumferential membrane in >10% of the cells. Overexpression was evaluated as positive for scores of 2+ or 3+ and as negative for scores of 0 or 1+ (Supplemental Figure S1) [39]. For nuclear Ki-67 expression, the percentage of positive cells among 100 cancer cells from three randomly selected fields of vision observed using a high-power lens was calculated.

### 2.5. TUNEL Assays

Cell death was assessed using the TUNEL (TdT-mediated dUTP nick end labeling) method and an In Situ Cell Death Detection Kit (Roche, #11684817910) following the manufacturer’s instructions. For the TUNEL staining of nuclei, the percentage of positive cells among at least 100 cancer cells from three randomly selected fields of vision observed using a high-power lens (×400) was calculated.

### 2.6. Patient Selection

Tumor samples from seventy-nine patients with EMPD were immunohistochemically assessed. All of the patients had been diagnosed and treated at the Department of Dermatology, Hokkaido University Hospital. The EMPD samples were obtained from patients whose ages ranged from 46 to 94 years (average, 74.59 years; male–female ratio, 44: 35). This research was approved by the institutional review board of Hokkaido University Hospital (#021-0220). The initial clinical stage and observation duration were retrieved from clinical data. The TNM stage was defined in accordance with the EMPD staging system proposed by Ohara et al. [2]. Primary tumors of greater than 4 mm in thickness or with lymphovascular invasion were defined as T2, and primary tumors that did not meet these criteria were defined as T1. The patients were classified into four stages: distant metastasis as stage IV, lymph node (LN) metastasis as stage III, advanced primary tumor as stage II, and early primary tumor as stage I [2].

### 2.7. Statistical Analyses

Quantitative data are described as mean ± standard deviation (SD). All statistical analyses were performed using Excel 2016 (Microsoft Corporation) and the Excel add-in software Statcel (OMS Ltd., Tokyo, Japan). The Student’s *t*-test was used to evaluate tumor volumes between the treatment groups and the control group. At least three independent experiments were carried out for statistical comparison. We used the Fisher’s exact probability test to assess pairwise comparisons among groups. Kaplan–Meier survival curves were calculated for the two or four groups (HER2-positive or -negative), and the log-rank test was used to compare disease-specific survival. *P* values of less than 0.05 were considered significant.

## 3. Results

### 3.1. HER-2-Targeted ADCs Regress the Tumor Growth of EMPD-PDXs

**Figure 2:**
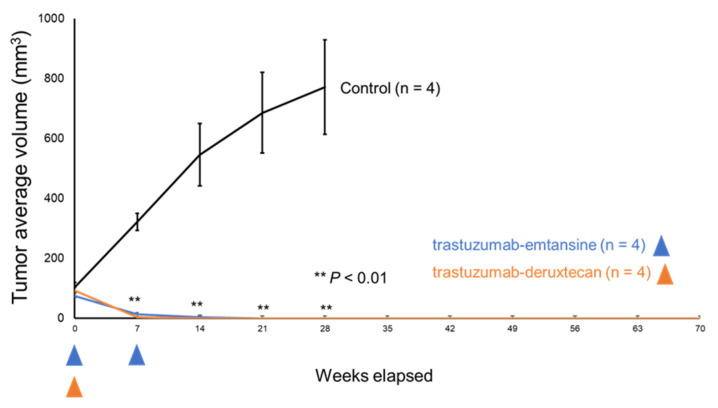
HER2-targeted ADCs suppress tumor growth in extramammary Paget’s disease patient-derived xenografts. Tumor-bearing NOD/Scid mice were randomized into three groups: no therapy (control, black line), trastuzumab emtansine (blue line), and trastuzumab deruxtecan (orange line). Tumor volume was calculated using the following formula: (long axis × short axis^2^)/2. The data are presented as means, with error bars representing the SD from the mean. The blue or orange arrowheads indicate the injection of trastuzumab emtansine or trastuzumab deruxtecan, respectively. ** *p* < 0.01 vs. control.

**Figure 3:**
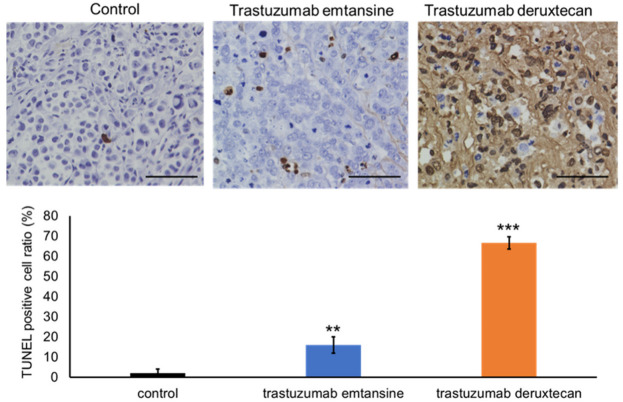
The positive ratio of TUNEL staining is markedly higher in the EMPD-PDX-H1 tumors treated with HER2-targeted ADC than in the untreated control tumors. EMPD tumors were treated with HER2-targeted ADCs (trastuzumab emtansine or trastuzumab deruxtecan). After 48 h, the tumors were extracted and were assessed by TUNEL staining. Upper: Representative images of TUNEL staining (scale bar = 100 µm). Lower: The ratio of TUNEL-positive cells was examined (columns, mean percentage of TUNEL-positive cells, n = 3, determinations based on examination of 100 tumor cells; bars, SD). ** *p* < 0.01, *** *p* < 0.001; compared to control tumors, by *t*-test.

**Figure 4:**
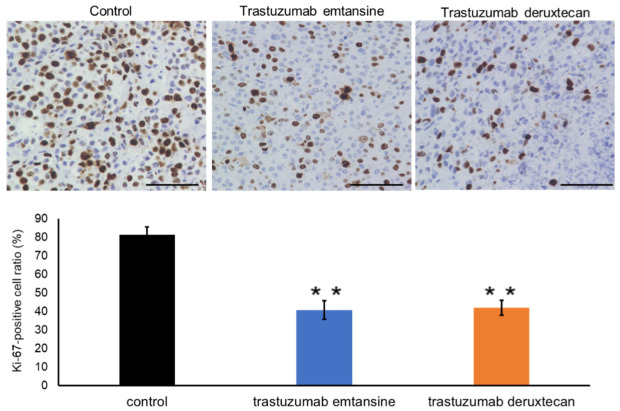
The ratio of Ki-67-positive cells is lower in the HER2-targeted ADC-treated cells than in the control tumor cells. EMPD tumors were treated with HER2-targeted ADCs (trastuzumab emtansine or trastuzumab deruxtecan). After 48 h, the tumors were extracted and were assessed by Ki-67 staining. Upper: Representative images of Ki-67 staining (scale bar = 100 µm).Lower: The ratio of Ki-67-positive cells was examined (columns, mean percentage of Ki-67-positive cells, n = 3, determinations based on examination of 100 tumor cells; bars, SD). ** *p* < 0.01; compared to control tumors, by *t*-test.

Previously, our group investigated the protein expression levels of HER2 in PDX tumor cells (EMPD-PDX-H1) [31]. Tumor cells in the EMPD-PDXs scored 1+ for HER2. Targeted gene mutation analysis using a comprehensive cancer panel (Qiagen) revealed pathogenic genomic DNA alterations in *ERBB2* (c.929C > T, p.S310F) in EMPD-PDXs [31]. Based on the gene mutation analysis, we conducted a therapeutic examination using HER2-targeted ADCs to examine whether EMPD-PDX-H1 harboring the pathogenic *ERBB2* mutation responds to such therapies. We administered intravenous injections of T-DM1 (15 mg/kg) once a week for two weeks or of T-DXd (10 mg/kg) once. The HER2-targeted ADCs (T-DM1 and T-DXd) were found to regress EMPD-PDX tumors remarkably in only seven days (Figure 2). At 14 days after the initial injections, the subcutaneous tumors were not palpable, suggesting that the PDX tumors had been eradicated. Further, we observed no recurrence of PDX-tumors for 10 weeks with either therapeutic. To examine the status of the treated cells, we extracted tumors 48 h after the intravenous administration of the drugs. The positive ratio of TUNEL staining was remarkably higher for the HER2-targeted ADC-treated EMPD-PDX-H1 tumors than for the untreated control tumors (Figure 3). Additionally, the extracted tumors revealed the ratio of Ki-67-positive cells to be lower in the HER2-targeted ADC-treated cells than in the control tumor cells (Figure 4). These histopathological results indicate that the HER2-targeted ADCs are highly effective and are able to kill EMPD cells promptly. The present results are consistent with a previous study using PDX tumors harboring pathogenic *ERBB2* mutations in other cancers [36].

### 3.2. HER2 Expression Correlates with Invasion and Disease-Specific Survival

**Figure 5:**
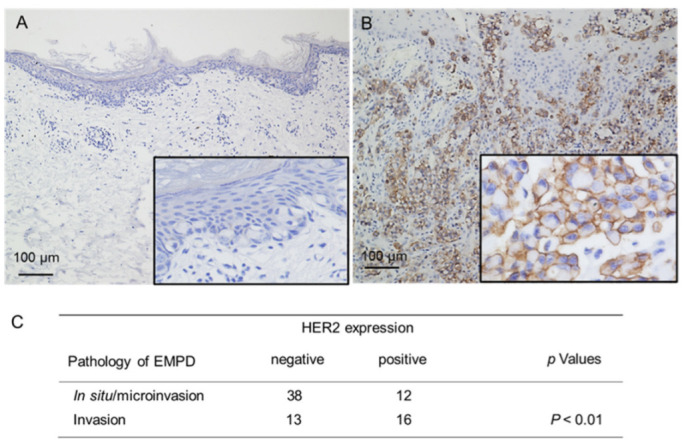
There is a significant correlation between positive HER2 immunostaining and the presence of invasive lesions. (**A**,**B**) Representative images of immunohistochemical staining for HER2; (**A**) in situ extramammary Paget’s disease (EMPD); (**B**) invasive EMPD. (**C**) The 79 cases were classified as HER2-negative or HER2-positive. HER2-negative is significantly more common in situ/microinvasive EMPD tumors than in invasive EMPD (*p* < 0.01, Fisher’s exact probability test).

**Figure 6:**
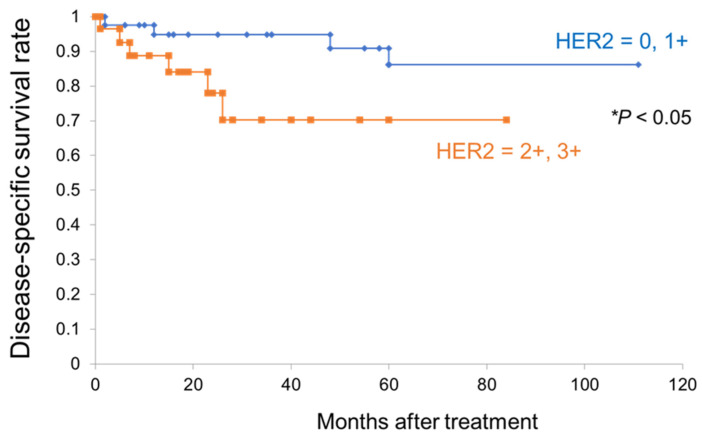
A significant correlation is observed between HER2-positive immunostaining and disease-specific survival. Kaplan–Meier curves to determine disease-specific survival for extramammary Paget’s disease patients are shown (blue: HER2-negative group (N = 51), orange: HER2-positive group (N = 28)) (*p* = 0.045, log-rank test).

Next, we immunohistochemically examined the expression levels of HER2 in EMPD clinical specimens. We assessed 79 specimens of primary EMPD skin lesions. The clinical information is summarized in Table 1. Of the specimens of in situ/microinvasive EMPD (50 cases), positive HER2 immunostaining was observed in 12 cases (24%: 3+ for 3 cases, 2+ for 9 cases). Of the specimens of invasive EMPD (29 cases), positive HER2 immunostaining was observed in 16 cases (55%: 3 + for 7 cases, 2 + for 9 cases) (Figure 5). Thus, there was a significant correlation between positive HER2 immunostaining and the presence of invasive lesions (*p* < 0.01). Our study included six cases of EMPD with lymph node metastasis; thus, we assessed the HER2 expression of six specimens of lymph node metastasis. In three out of six such cases (50%), the expression levels of HER2 in the lymph node metastases were lower than those in the corresponding primary tumors (Supplemental Figure S2). Additionally, no significant correlation was found between the HER2 status of the primary skin lesions and lymph node metastasis (*p* = 0.44, Table 1). We also investigated the correlations between HER2 expression and disease-specific survival. The disease-specific survival was significantly worse for cases with positive HER2 immunostaining (28 cases) than for cases with negative HER2 immunostaining (51 cases) (*p* = 0.045, Figure 6). These results are consistent with a previous study by Tsutsumida et al. [38], in which the 5-year survival rate was significantly worse in cases with invasive EMPD than in cases with in situ/microinvasive EMPD. Further, we assessed the prognosis (disease-specific survival) based on HER2 status as well as EMPD pathology. The patients were divided into four groups: 1: microinvasive/invasive cases with positive HER2 immunostaining (*N* = 19), 2: in situ cases with positive HER2 immunostaining (*N* = 9), 3: microinvasive/invasive cases with negative HER2 immunostaining (*N* = 26), and 4: in situ cases with negative HER2 immunostaining (*N* = 25). The disease-specific survival was significantly worse for microinvasive/invasive cases with positive HER2 immunostaining (19 cases) than for in situ cases with negative HER2 immunostaining (25 cases) as well as for microinvasive/invasive cases with negative HER2 immunostaining (26 cases) (*p* = 0.04, *p* = 0.021, respectively, Supplemental Figure S3). The disease-specific survival was worse for microinvasive/invasive cases with positive HER2 immunostaining than for in situ cases with positive HER2 immunostaining; however, the difference was not significant (*p* = 0.07). Concerning the clinical stages and HER2 status, HER2 expression was higher in progressive clinical stages than in early clinical stages; however, the correlation was not significant (Table 1).

## 4. Discussion

In the present study, treatments with trastuzumab emtansine (T-DM1) or trastuzumab deruxtecan (T-DXd) significantly regressed tumors in EMPD patient-derived xenografts (PDXs) harboring the pathogenic *ERBB2* mutation. Two HER2-targeted ADCs—T-DM1 and T-DXd—were found to eradicate EMPD tumors in two weeks (Figure 2), in contrast to trastuzumab monotherapy, which is found to only suppress tumor growth [31]. Further, we observed no recurrence of PDX-tumors for 10 weeks following the HER2-targeted ADC therapies. These results suggest that the HER2-targeted ADC therapeutics are more potent against EMPD than trastuzumab monotherapy is.

More than 15 case reports have addressed HER2-targeted therapies against EMPD [14,15,16,17,18,19,20,21,22,23,24,25,26,27,28,29,30]. Most of the cases are EMPD with HER2 overexpression evaluated by IHC. Previously, our group established an EMPD-PDX model harboring the *ERBB2* S310F mutation without obvious HER2 protein overexpression [31]. This mutation has been described as an extracellular domain mutation of HER2, and it is the most common pathogenic mutation of *ERBB2*. *ERBB2* S310F mutations have been speculated to result in HER2 activation via elevated C-terminal tail phosphorylation or via covalent dimerization mediated by intermolecular disulfide bond formation [32]. A number of proteins regulating cytoskeletal dynamics and cell motility were found to be prominently hyperphosphorylated in *ERBB2* S310F-expressing cells. Previous studies reported that the clinical efficacy of HER2-targeted ADCs in lung cancers depends on *ERBB2* mutations or amplification and not on the quantity of HER2 protein expression [36,40]. These studies suggest that HER2 receptor hyperactivation through gene mutation or amplification, rather than its overexpression, is a key mechanism underlying the internalization of the receptor–ADC complex and the consequent efficacy of ADCs. Two studies have addressed the *ERBB2* S310F mutation in EMPD, and they indicate that 12.5% of EMPD cases harbor the *ERBB2* S310F mutation [41]. In the future, if any clinical evidence is obtained for the efficacy of HER2-targeted ADCs against advanced EMPD with the *ERBB2* S310F mutation, then *ERBB2* gene mutation analysis (especially of the S310F mutation) should be evaluated in advanced EMPD to explore optimal treatment selection.

In several previous studies on HER2 positivity in EMPD, the overexpression of HER2 was found to correlate with the disease progression of EMPD [8]. The present study also shows the positive expression of HER2 to be significantly more frequent in patients with invasive EMPD than in those with in situ/microinvasive EMPD, which suggests that HER2-positive tumor cells have increased invasive potential. Thus, the ratio of HER2-positive cases could be higher in advanced EMPD cases than in other EMPD cases. On the other hand, several studies found no significant correlation between HER2 status and Ki-67 immunoreactivity [42,43]. Another observation of this study is that the expression of HER2 could be down-regulated in metastatic lymph nodes compared to that expression in the corresponding primary skin tumors. These results are consistent with a previous study by Tanaka et al. [44], who speculated that the heterogeneity of EMPD tumors was one reason for the discrepancy in HER2 expression.

In addition to the small sample size, a limitation of our research is that we have established only one EMPD-PDX model [31]. In the future, we will need to establish other EMPD-PDX models and EMPD cell cultures to confirm the present results.

## 5. Conclusions

Our results revealed in vivo tumor regression through HER2-targeted ADCs in EMPD-PDX models. HER2-targeted ADCs could be effective treatments for *ERBB2*-mutated EMPD tumors and possibly also for HER2-overexpressed cases.

## Figures and Tables

**Table 1:** Clinical summary of the 79 EMPD patients.

		Total Number	HER2- Negative	HER2- Positive	*p* Value
Cases		79	51	28	
Age	Range	46–94			
	(Mean)	−74.59			
Sex	Male	44 (55.7%)	27	17	0.33
	Female	35 (44.3%)	24	11	
Primary site	Genital/anal	74	46	28	0.17
	Axillary	4	4	0	
	Inguinal	1	1	0	
Clinical stage	In situ	34 (43.1%)	25	9	0.07
	I	5 (6.3%)	4	1	
	II	31 (39.2%)	18	13	
	III	6 (7.6%)	3	3	
	IV	3 (3.8%)	1	2	
EMPD pathology	In situ	34 (43.1%)	25	9	0.006
	Microinvasive	16 (20.2%)	13	3	
	Invasive	29 (36.7%)	13	16	
Lymph node metastasis	+	6 (7.6%)	3	3	0.44
	-	73 (92.4%)	48	25	
Outcome	Alive	70 (88.6%)	49	21	0.008
	Dead	9 (11.4%)	2	7	

## Data Availability

Data supporting the reported results can be obtained from the corresponding author.

## Supplementary Materials

The following supporting information can be downloaded at: https://www.mdpi.com/article/10.3390/cancers14143519/s1, Figure S1: Representative images of HER2 immunostaining; Figure S2: Levels of HER2 expression in lymph node metastases are not identical to those in the corre-sponding primary skin tumors; Figure S3: A significant correlation is observed between HER2-positive immunostaining and disease-specific survival. Table S1: EMPD Cases treated with HER2-targeted therapies.

## Abbreviation

ADC	antibody–drug conjugate
EMPD	extramammary Paget’s disease
HER2	human epidermal growth factor receptor 2
IHC	immunohistochemistry
LN	lymph node
PDX	patient-derived xenograft
T-DM1	trastuzumab emtansine
T-DXd	trastuzumab deruxtecan
TUNEL	TdT-mediated dUTP nick end labeling
