# Supplementary material for: HER2-Targeted Antibody–Drug Conjugates Display Potent Antitumor Activities in Preclinical Extramammary Paget’s Disease Models: In Vivo and Immunohistochemical Analyses
Source: Cancers (Basel). 2022 Jul 20;14(14):3519. doi: 10.3390/cancers14143519 (PMC9322551; doi:10.3390/cancers14143519)
Supplement: Supplementary file 1 [file cancers-14-03519-s001.zip › cancers-1773528-supplementary.pdf]

Supplemental information

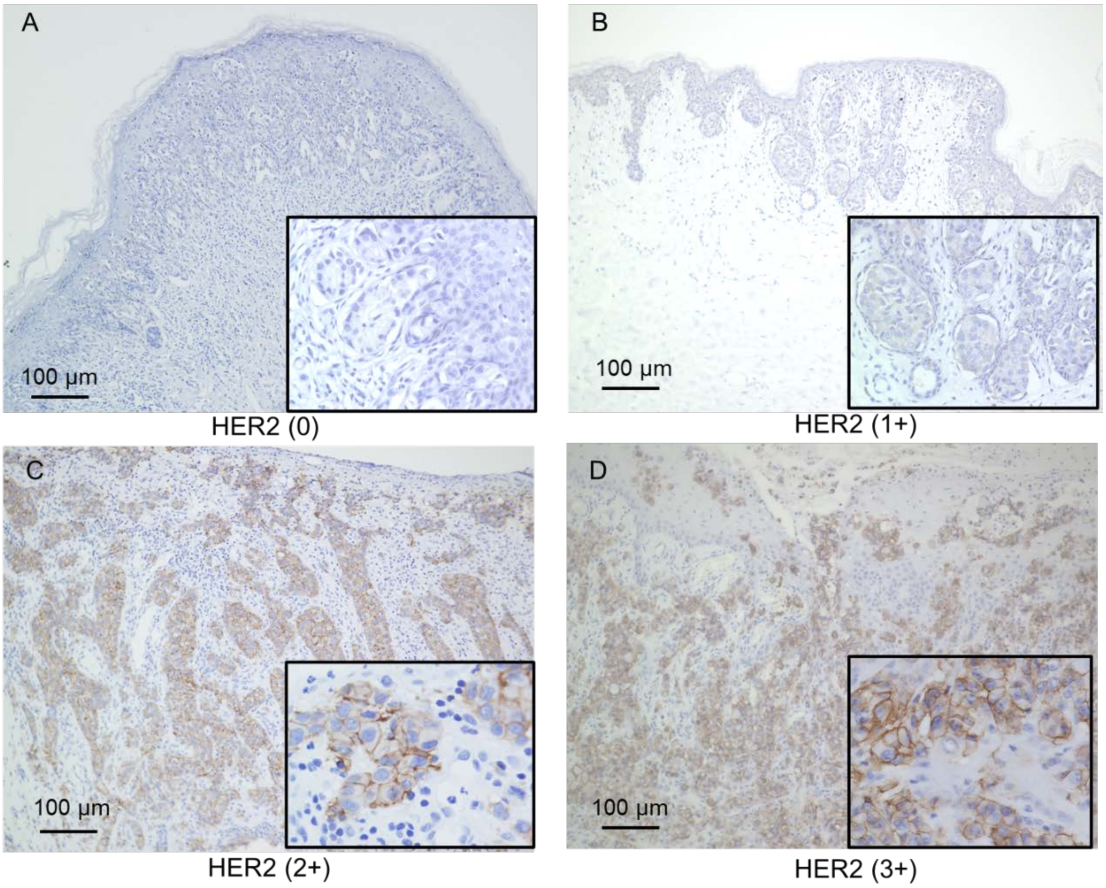

Supplemental Figure S1. Representative images of HER2 immunostaining.

A representative image is shown for each score (scale bar = 100 µm).

(A) HER2 (0); (B) HER2 (1+); (C) HER2 (2+); (D) HER2 (3+).

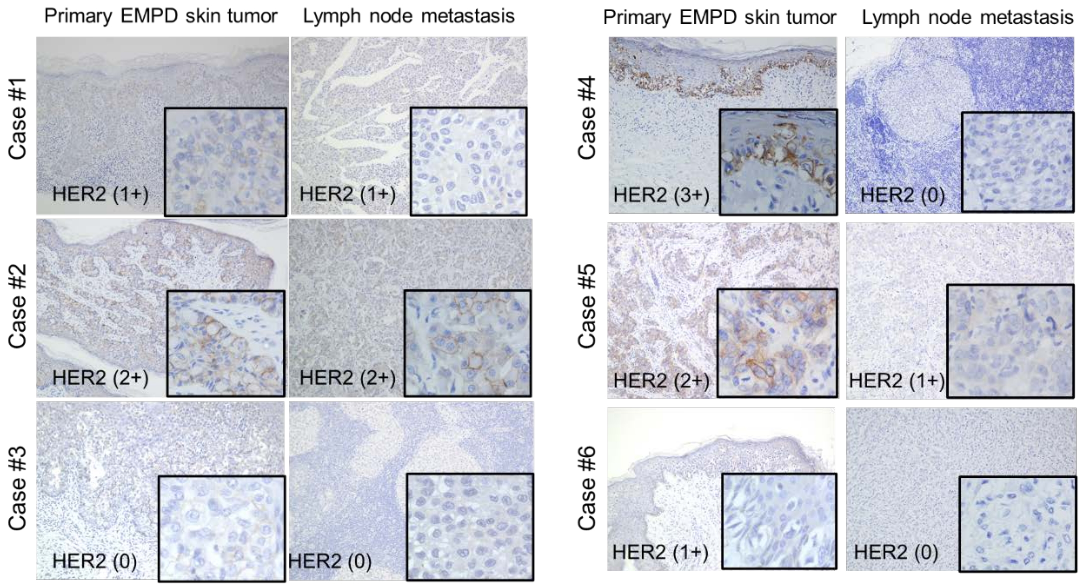

Supplemental Figure S2. Levels of HER2 expression in lymph node metastases are not identical to those in the corresponding primary skin tumors.

The present study includes six EMPD cases with lymph node metastasis. We assessed the HER2 expression of the primary sites and lymph node metastasis in these cases. Three of the six cases show no difference in HER2 expression between the primary site and the lymph node metastasis (left column, Cases #1–#3). The other three cases show lower HER2 expression in lymph node metastasis than in the primary site (right column, Cases #4–#6).

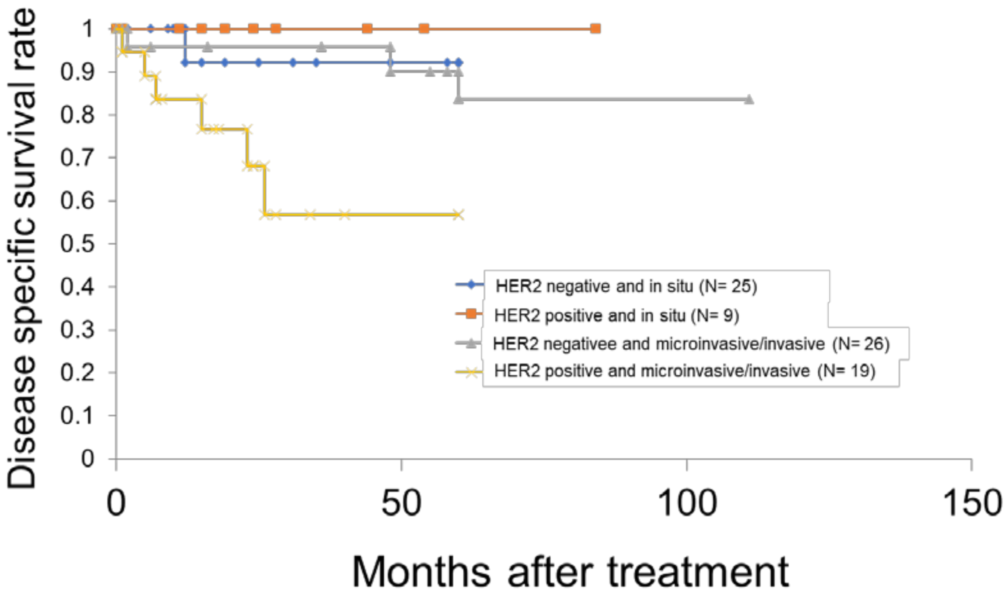

Supplemental Figure S3. A significant correlation is observed between HER2-positive immunostaining and disease-specific survival.

We assessed the prognosis (disease-specific survival) based on HER2 status as well as EMPD pathology. The patients were divided into four groups: 1: microinvasive/invasive cases with positive HER2 immunostaining (N = 19), 2: *in situ* cases with positive HER2 immunostaining (N = 9), 3: microinvasive/invasive cases with negative HER2 immunostaining (N = 26), and 4: *in situ* cases with negative HER2 immunostaining (N = 25). Kaplan–Meier curves of disease-specific survival for the patients are shown. The disease-specific survival is significantly worse for microinvasive/invasive cases with positive HER2 immunostaining than for *in situ* cases with negative HER2 immunostaining or microinvasive/invasive cases with negative HER2 immunostaining (p = 0.04, p = 0.021, respectively, log-rank test).

Supplemental figure table S1; EMPD Cases treated with HER2-targeted therapies

Reference	No. of patients	Treatment	HER2 IHC	HER2 mutation	Response	Outcome (months)
13. Vornicova	1	lapatinib	negative	S310F	PR	PFS:12＋
14. Karam	1	trastuzumab	3＋	unknown	PR	PFS: 12
15.Takahagi	1	trastuzumab + paclitaxel	positive	unknown	PR	PFS: 17, OS: 25
16. Hanawa	1	trastuzumab + paclitaxel	3＋	unknown	PR	PFS: 13
17. Wakabayashi	1	trastuzumab	3＋	unknown	PR	PFS: 12+
18. Yoshimura	1	trastuzumab + taxianes	3＋	unknown	PR	PFS:4
19. Barth	1	trastuzumab	3＋	none	CR	PFS: 12
20. Zhang	1	trastuzumab	overexpression	unknown	PR	PFS:15＋
21. Shin	1	trastuzumab＋docetaxel＋carboplatin followed by lapatinib and T-DM1	overexpression	unknown	CR	PFS: almost 12＋
22. Watanabe	1	trastuzumab ＋ docetaxel ＋ pertuzumab	overexpression	unknown	PR	PFS:12
23. Ichiyama	1	trastuzumab + paclitaxel	positive	unknown	PR	PFS: 24+
24. Nordmann	1	trastuzumab ＋ carboplatin	3＋	S310F	near CR	PFS: 7.5
25. Lu	2	trastuzumab+paclitaxel＋cisplatin	3＋	none	CR	PFS:17
		trastuzumab + paclitaxel	2＋	genetic heterogeneity	SD	PFS:5
26. Sekiguchi	4	trastuzumab + paclitaxel	3＋:2, 2＋:2	unknown	PR(1/4), PD(3/4)	MPFS: 5.3, MOS: 12.0
27. Bartoletti	4	trastuzumab + paclitaxel	positive	unknown	CR(2/4), PR(2/4)	MDOR: 10 (2–34)
28. Bruce	1	trastuzumab	positive	unknown	CR	PFS:12＋
29. Guo	1	pyrotinib	unknown	R678Q/S310Y/S310F	PR	PFS: 2＋

CR, complete response; PR, partial response; SD, progressive disease; PFS, progression-free survival; MPFS, median progression-free survival; OS, overall survival; MOS, median overall survival; ＋, ongoing response; MDOR, median duration of response
